# Visual impairment as a predictor for deterioration in functioning: the Leiden 85-plus Study

**DOI:** 10.1186/s12877-022-03071-x

**Published:** 2022-05-06

**Authors:** ERJ Verbeek, YM Drewes, J Gussekloo

**Affiliations:** 1 Department of Internal Medicine, section Gerontology and Geriatrics, Leiden University Medical Center, Leiden, the Netherlands; 2 Department of Internal Medicine, section Gerontology and Geriatrics, Leiden University Medical Center, Leiden, the Netherlands; 3 Department of Public Health and Primary Care and Department of Internal Medicine, section Gerontology and Geriatrics, Leiden University Medical Center, Leiden, the Netherlands

**Keywords:** Older adults, Visual impairment, Cohort study, Prediction, Functioning, Quality of life, Mortality

## Abstract

**Background:**

Visual impairment frequently occurs amongst older people. Therefore, the aim of this study was to investigate the predictive value of visual impairment on functioning, quality of life and mortality in people aged 85 years.

**Methods:**

From the Leiden 85-plus Study, 548 people aged 85 years were eligible for this study. Visual acuity was measured at baseline by Early Treatment Diabetic Retinopathy Study charts (ETDRS). According to the visual acuity (VA) three groups were made, defined as no (VA > 0.7), moderate (0.5 ≤ VA ≤ 0.7) or severe visual impairment (VA < 0.5). Quality of life, physical, cognitive, psychological and social functioning were measured annually for 5 years. For mortality, participants were followed until the age of 95.

**Results:**

At baseline, participants with visual impairment scored lower on physical, cognitive, psychological and social functioning and quality of life (*p* < 0.001). Compared to participants with no visual impairment, participants with moderate and severe visual impairment had an accelerated deterioration in basic activities of daily living (respectively 0.27-point (*p* = 0.017) and 0.35 point (*p* = 0.018)). In addition, compared to participants with no visual impairment, the mortality risk was 1.83 (95% CI 1.43, 2.35) for participants with severe visual impairment.

**Discussion:**

In very older adults, visual impairment predicts accelerated deterioration in physical functioning. In addition, severely visually impaired adults had an increased mortality risk. A pro-active attitude, focussing on preventing and treating visual impairment could possibly contribute to the improvement of physical independence, wellbeing and successful aging in very old age.

**Supplementary Information:**

The online version contains supplementary material available at 10.1186/s12877-022-03071-x.

## Introduction

Worldwide, a total of 2.2 billion have impaired vision [1]. Prevalence and incidence are increasing with age. Aging is one of the major risk factors for vision loss followed by smoking [2]. Age-related macular degeneration is the leading cause of blindness, followed by diabetic retinopathy, glaucoma and cataract. For these diseases, effective strategies exist to delay or prevent the visual impairment or blindness from occurring [3]. However, in older people visual problems are frequently underreported, overlooked or dismissed. Consequently, the burden associated with diminished vision is significant [3, 4].

Visual impairment can affect daily functioning, social participation and cognitive state, highlighting the importance of improving health services to promote healthy aging [5–8]. Impaired vision is associated with an increased fall risk [9], comprised mobility [10], poorer quality of life [11], isolation often resulting in depression [12], cognitive dysfunction [13] and higher mortality rates [14]. However, the association between visual impairment and the changes in level of functioning in very old adults is still unknown.

Therefore, in this research the predictive value of visual impairment on functioning, quality of life and mortality was studied, based on the data from the Leiden 85-plus Study. Three hypotheses were tested for older people with visual impairment: (i) they have a lower level of functioning and quality of life at baseline, (ii) they deteriorate faster in functioning and quality of life and (iii) visual impairment is associated with higher mortality risk.

## Materials and methods

### Population characteristics

The Leiden 85-plus Study, an observational population-based prospective cohort study, examined health, functioning and well-being in very old adults. Included were all people from Leiden, the Netherlands, who turned 85 between 1 September 1997 and 1 September 1999. Details are specified in a previous study [15]. Participants were visited annually by a research nurse, at their current place of residence, for interviews and performance tests. Furthermore, the participants’ medical history was acquired from the general practitioner, pharmacy records and/or the nursing home physician. Common chronic diseases were included such as arthritis, obstructive pulmonary disease, cerebrovascular accident, myocardial infarction, Parkinson’s disease, malignancy and diabetes mellitus. Subsequently, there was a 5-year follow-up for morbidity and level of functioning. For mortality, participants were followed until 95 years of age. All participants gave informed consent. In the case of severe cognitive impairment, this consent was obtained from a guardian. Furthermore, the study was approved by the Medical Ethics Committee of the Leiden University Medical Centre.

### Determinant

At baseline, visual acuity (VA) was assessed with objective measurements, using the Early Treatment Diabetic Retinopathy Study charts (ETDRS), at a distance of three meters for the left eye, the right eye and both eyes simultaneously. Participants were asked to wear their corrective glasses during the assessment. Visual acuity was reported on decimal scale. Participants with missing data were excluded. Participants were stratified into three groups at baseline. No visual impairment was defined as a visual acuity of more than 0.7, moderate visual impairment as a visual acuity from 0.5 to 0.7 and severe visual impairment as a visual acuity of less than 0.5 [16].

### Outcomes

#### Functional status

All participants were followed for 5 years or until their death and their functional status was measured annually. Functional status was divided in four subcategories: (i) physical, (ii) cognitive (iii) psychological and (iv) social functioning.

##### Physical functioning

was assessed with the Groningen Activity Restriction Scale (GARS), a non-disease specific instrument to measure disability in basic activities of daily living (BADL) and instrumental activities of daily living (IADL) [17]. The GARS contains nine questions regarding BADL and nine questions regarding IADL [18]. The minimal score for both BADL and IADL is 9 and the maximal score is 36. A score of 9 represents optimal function. For participants who had severe cognitive impairment (Mini-Mental State Examination below 19), information was obtained from a proxy.

##### Cognitive functioning

was measured with the Mini-Mental State Examination (MMSE) with scores varying from 0 to 30 (30 = optimal cognitive function) [19].

##### Psychological functioning

was assessed by measuring depressive symptoms with the 15-item Geriatric Depression Scale (GDS-15), with a score from 0 to 15, in which higher scores indicated more depressive symptoms [20]. The GDS-15 was administered only in participants with a MMSE score > 18 points.

##### Social functioning

was quantified with the De Jong Gierveld Loneliness Scale (DJG), an 11-item questionnaire combining emotional loneliness (six items) and social loneliness (five items) specifically developed for use in older populations, with an outcome from 0 to 11 (0 = not lonely) [21] and was solely administered in participants with a MMSE score > 18 points.

#### Quality of life

Quality of life was assessed with the Cantril’s Ladder, a visual analogue scale on perceived quality of life, varying from 0 to 10 (10 = extremely satisfied) [22].

#### Mortality

Mortality data was obtained from municipal registry recorded between the start of the study and Feb. 1, 2008.

### Statistical analysis

Differences in baseline characteristics between the groups according to visual impairment were analysed with the Linear by Linear Association for categorical variables and the Jonckheere-Terpstra Test for continuous variables. Prospectively, differences between changes in functional status and quality of life in these groups were estimated using linear mixed models and presented as (predicted) means with standard errors. The mixed models included a term for time, vision status (severe, moderate or no visual impairment) and a term for interaction between time and vision status. In subjects with no visual impairment, the effect of time on the level of functioning reflects the annual changes in functioning. The interaction between the functioning levels and time, in an individual with impaired vision, represents the additional annual change in functioning. The time till death was estimated using Kaplan Meier curves and compared using a log-rank test. Mortality hazard ratios (HR) and corresponding 95% confidence intervals (CI) were calculated in a Cox proportional hazards model.

As a sensitivity analysis different cut-off values for visual impairment were used. Primarily, participants were ranked in three equal groups according to 33% tertiles (high, medium and low vision). Additionally, participants were categorized in three groups following the International Classification of Diseases and Related Health Problems (ICD-10) guidelines, with a visual acuity below 0.33 defined as low vision and 0.5 or higher as normal vision [23]. Lastly, the cross-sectional correlation between vision and functioning was examined as the effect of a decrease per 0.1 point in vision on the level of functioning. Statistical analysis was executed with IBM SPSS statistics version 24.0.

## Results

### Study population

A total of 705 inhabitants were eligible for the study. Before enrolment in the Leiden 85-plus Study, 14 persons died and 92 declined to participate because of several reasons, such as no interest, no time, too nervous or anxious, too tired or ill or being against surveys in general [15]. Of the 599 subjects, 51 participants had no or an incomplete visual assessment due to severe illness or unknown reasons. The demographics of these participants were similar compared with the group with complete visual assessment, however, a history of cerebrovascular accident and severely cognitive impairment were present more often. Individuals with missing data were excluded from this study. Therefore, baseline data were available of 548 participants.

### Baseline characteristics

Table 1 presents the baseline characteristics. At baseline, 215 (39.2%) participants had no visual impairment, 215 (39.2%) moderate visual impairment and 118 (21.5%) severe visual impairment. Participants with severe visual impairment were less often men (27.1 versus 31.6% for moderate impairment and 39.1% for no impairment, *p*_trend_ = 0.021), less often living independently (71.2 versus 84.2% for moderate impairment and 91.6% for no impairment, *p*_trend_ < 0.001) and less often having a high income (39.7 versus 48.4% for moderate impairment and 57.3% for no impairment, *p*_trend_ = 0.002). Moreover, the prevalence of diabetes and severe cognitive impairment was higher in participants with severe visual impairment (respectively p _trend_ = 0.006 and < 0.001). Arthritis and osteoarthritis are less prevalent in this group (24.6 versus 31.3% in moderate group and 38.8% in no impairment group, *p*_trend_ = 0.007). Between the groups, there was no difference in fall history, however, hip fractures occurred significantly more in the severe visually impaired group (*p*_trend_ = 0.005). The level of physical, cognitive, psychological and social functioning at baseline was significantly lower for the groups with moderate and severe visual impairment (all p _trend_ < 0.001). Furthermore, a difference was found for quality of life when comparing the three groups (p _trend_ < 0.001).

**Table 1:** Baseline characteristics of the participants (*n* = 548) depending on degree of visual impairment

	**All *n* = 548**	**No visual impairment *n* = 215**	**Moderate visual impairment *n* = 215**	**Severe visual impairment *n* = 118**	P-Value*
Demographics and health (No., %)					
Male	184 (33.6)	84 (39.1)	68 (31.6)	32 (27.1)	**0.021**
Living arrangements: Independently	462 (84.3)	197 (91.6)	181 (84.2)	84 (71.2)	** < 0.001**
Education > elementary school˚	193 (35.3)	88 (40.9)	66 (30.7)	39 (33.3)	0.089
High income˚	271 (50.0)	122 (57.3)	103 (48.4)	46 (39.7)	**0.002**
Chronic diseases					
Arthritis/osteoarthritis ˚	179 (32.8)	83 (38.8)	67 (31.3)	29 (24.6)	**0.007**
Obstructive pulmonary disease	63 (11.5)	32 (14.9)	17 (7.9)	14 (11.9)	0.227
Cerebrovascular accident ˚	44 (8.1)	18 (8.4)	15 (7.0)	11 (9.4)	0.866
Myocardial infarction ˚	56 (10.3)	20 (9.3)	23 (10.7)	13 (11.0)	0.598
Parkinson’s disease	11 (2.0)	5 (2.3)	2 (0.9)	4 (3.4)	0.704
Malignancy ˚	100 (18.3)	42 (19.5)	35 (16.3)	23 (19.8)	0.899
Diabetes mellitus ˚	79 (14.5)	26 (12.1)	24 (11.2)	29 (24.8)	**0**.**006**
Severe cognitive impairment (MMSE < 19)˚	89 (16.3)	18 (8.4)	38 (17.7)	33 (28.2)	** < 0.001**
History fall˚	93 (17.2)	40 (18.9)	32 (15.0)	21 (18.1)	0.916
Hip fracture˚	33 (6.1)	6 (2.8)	15 (7.0)	12 (10.3)	**0.005**
Functioning and quality of life (median, IQR):					
Physical functioning					
BADL (*n* = 547)	10.0 (9.0–14.0)	9.0 (9.0–11.0)	10.0 (9.0–13.5)	11.0 (9.0–18.3)	** < 0.001**
IADL (*n* = 547)	18.0 (12.0–25.0)	15.0 (11.0–21.0)	18.0 (12.8–25.0)	24.0 (16.0–33.0)	** < 0.001**
Cognitive functioning: MMSE	26.0 (23.0–28.0)	27.0 (25.0–29.0)	26.0 (22.0–28.0)	24.0 (19.0–28.0)	** < 0.001**
Psychological functioning: GDS (*n* = 475)^#^	2.0 (1.0–3.0)	1.0 (0.0–2.0)	2.0 (1.0–4.0)	2.0 (1.0–4.0)	** < 0.001**
Social functioning: DJG (*n* = 476)^#^	1.0 (0.0–3.0)	0.0 (0.0–2.0)	1.0 (0.0–3.0)	2.0 (0.0–4.0)	** < 0.001**
Quality of Life: Cantril (*n* = 521)	8.0 (7.0–9.0)	8.0 (7.0–9.0)	8.0 (7.0–9.0)	7.0 (6.0–8.0)	** < 0.001**

*BADL* Basic Activities Daily Living (range 9–36); *IADL* Instrumental Activities Daily Living (range 9–36); *MMSE* Mini Mental State Examination (range 0–30); *GDS* Geriatric Depression Scale (range 0–15); *DJG* De Jong Gierveld Loneliness Scale (range 0–11); *Cantril* Cantril’s ladder (range 0–10); *IQR* Interquartile Range. The median and interquartile ranges are provided when continuous variables have an asymmetric distribution. For categorical variables percentages are presented. *P Value for between group comparison with regard to vision, measured with *P for trend*: for categorical data with Linear-by-Linear Association, for continuous data with Jonckheere-Terpstra Test. ˚ Missing data for specific variables, according to three groups (severe – moderate – no impairment): 0–7 missing. ^#^Assessed only in participants with MMSE > 18

### Changes in functional status and quality of life over time

**Fig. 1:**
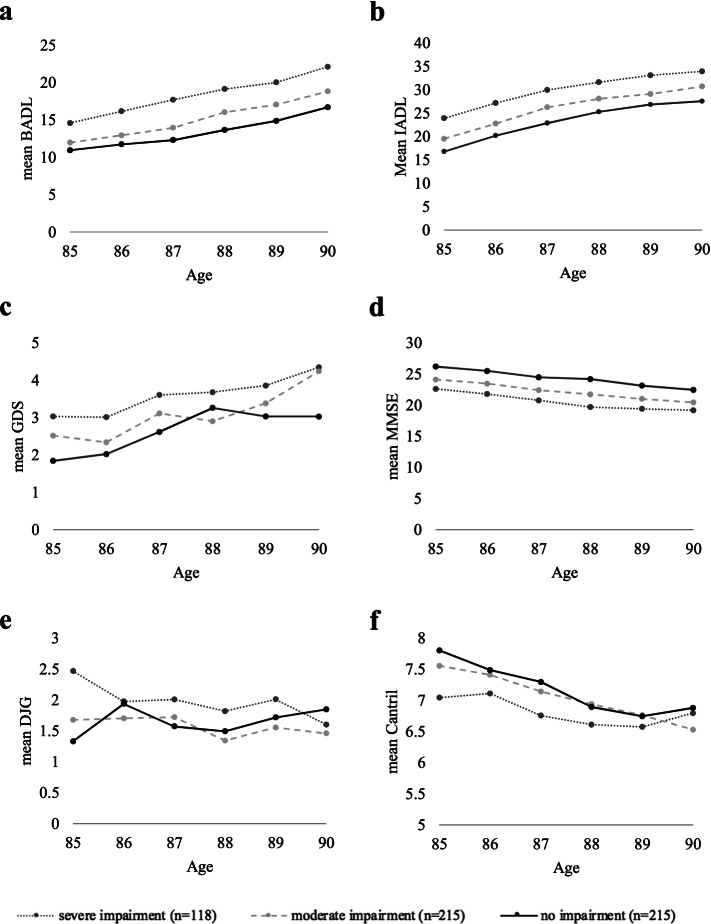
Effects of visual impairment on functional status and quality of life over time. Physical, cognitive and social functioning status were measured over time, during the 5 year follow-up period. Mean scores were compared for three groups according to the level of visual impairment. **a** Physical functioning: BADL. **b** Physical functioning: IADL. **c** Depression: GDS. **d** Cognition: MMSE. **e** Social functioning: DJG. **f** Quality of Life: Cantril

Table 2a describes the effect of visual impairment on the functional status and quality of life over time and results are visually represented in Fig. 1. Primarily, the basic annual change over time for the group with no visual impairment was determined in the linear mixed model analysis. A linear correlation was found between the level of functioning over time. For all variables this basic annual deterioration was statistically significant (at most *p* = 0.008). In participant with no visual impairment there was also an annual decrease in quality of life of 0.22 points on the Cantril’s Ladder (*p* < 0.001). Furthermore, compared to the participants with no visual impairment, the group with severe visual impairment had an additional annual deterioration in BADL of 0.35 points (*p* = 0.018, SE 0.146) and an additional annual change in quality of life of 0.12 points on the Cantril’s Ladder (*p* = 0.009,SE 0.048). In participants with moderate visual impairment an additional annual deterioration in BADL of 0.27 points was found (*p* = 0.017, SE 0.112) but not in quality of life. There was no significant additional annual change established for the other subcategories of functioning.

**Table 2:** Effect of visual impairment on changes in functioning and quality of life over time

	**Basic annual change**			**Additional annual change**					
	*No visual impairment*			*Moderate visual impairment*			*Severe visual impairment*		
	β_1_	SE	*P Value*	β_2_	SE	*P Value*	β_2_	SE	*P Value*
**a. All participants (*n* = 548)**									
BADL	1.10	0.076	** < 0.001**	0.27	0.112	**0.017**	0.35	0.146	**0.018**
IADL	2.24	0.079	** < 0.001**	0.04	0.117	0.735	-0.12	0.152	0.430
MMSE	-0.74	0.051	** < 0.001**	-0.02	0.076	0.830	-0.02	0.099	0.820
GDS	0.30	0.039	** < 0.001**	0.01	0.058	0.891	-0.05	0.079	0.566
DJG	0.08	0.029	**0.008**	-0.10	0.043	**0.023**	-0.14	0.059	**0.015**
Cantril	-0.22	0.024	** < 0.001**	0.01	0.035	0.676	0.12	0.048	**0.009**
**b. Participants with BADL = 9 (*n* = 256)**									
BADL	1.06	0.089	** < 0.001**	0.05	0.139	0.704	0.21	0.207	0.321
IADL	2.43	0.111	** < 0.001**	0.12	0.174	0.473	0.260	0.260	0.318
MMSE	-0.78	0.064	** < 0.001**	0.15	0.100	0.139	1.12	0.150	0.408
GDS	0.27	0.047	** < 0.001**	0.03	0.073	0.662	0.08	0.116	0.503
DJG	0.06	0.033	0.069	-0.06	0.051	0.248	-0.02	0.081	0.833
Cantril	-0.24	0.028	** < 0.001**	0.00	0.043	0.911	0.14	0.068	**0.040**
**c.** **Participants with BADL > 9 (*n* = 291)**									
BADL	1.17	0.129	** < 0.001**	0.48	0.178	**0.007**	0.41	0.209	0.052
IADL	1.93	0.109	** < 0.001**	0.08	0.150	0.585	-0.13	0.177	0.462
MMSE	-0.71	0.084	** < 0.001**	-0.20	0.116	0.085	-0.14	0.137	0.315
GDS	0.34	0.067	** < 0.001**	-0.02	0.094	0.850	-0.14	0.112	0.214
DJG	0.11	0.053	**0.039**	-0.15	0.076	**0.042**	-0.25	0.091	**0.006**
Cantril	-0.20	0.042	** < 0.001**	0.01	0.059	0.814	0.10	0.070	0.167

*SE* Standard error* P* Values were estimated by analysis of linear mixed models, significant when *P* Value < 0.05; function of the linear mixed model according to: y = α + βx. β firstly represents the basic annual change over time without impairment; and secondly the additional annual change for people with visual impairment.. β is given with corresponding SE

Table 2b and c illustrate the results of the in-depth analysis were participants were stratified according to their baseline level of functioning. Participants without physical impairment, were defined as a BADL score = 9 (*n* = 256), and participants with physical impairment, were defined as a BADL score > 9 (*n* = 291). Comparable to the overall analysis, a significant correlation was found between the level of functioning over time in both groups. In participants without physical impairment, an additional annual decline in quality of life of 0.14 points (*p* = 0.040, SE 0.068) was found for the people with severe visual impairment. For participants with physical impairment, a significant additional annual deterioration in BADL of 0.48 points (*p* = 0.007, SE 0.178) was found for the people with moderate visual impairment and of 0.41 points (*p* = 0.052, SE 0.209) for the people with severe visual impairment. This result was not observed in the group without physical impairment at baseline. In both groups, there were no other relevant significant additional annual change established for the other subcategories of functioning.

### Sensitivity analysis

In our sensitivity analysis similar trends were found by using different cut-off values for visual impairment. Results are presented in Additional file 1.

### Mortality

**Fig. 2:**
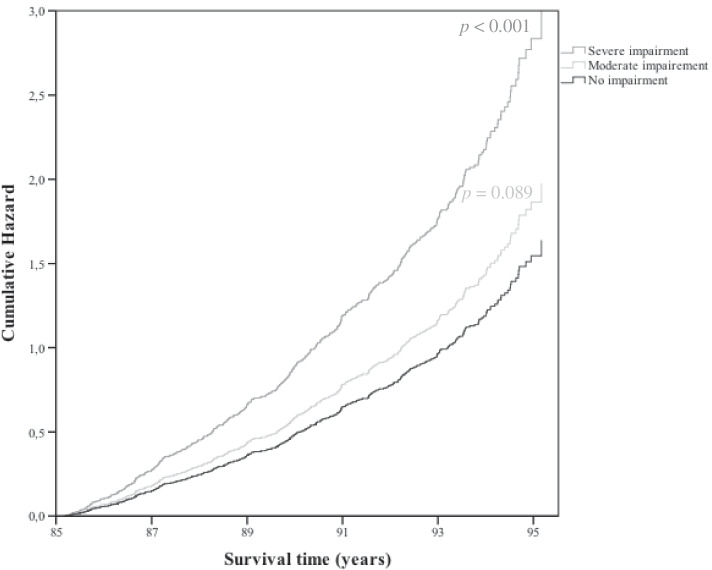
Cumulative Hazard ratios over a 10-year follow-up, according to visual status

The 10-year mortality risk increased from 1.21 (*p* = 0.089, 95% CI 0.97—1.50) in the group with moderate impaired vision to 1.83 (*p* < 0.001, 95% CI 1.43—2.35) in the group with severe impaired vision. The cumulative hazard ratios are depicted in Fig. 2.

## Discussion

In this population-based study, the relation between visual impairment and physical, cognitive and social functioning, quality of life and mortality was investigated, using the data from the Leiden 85-plus Study. At baseline, in older persons aged 85 years, moderate and severe visual impairment was associated with lower levels of physical, cognitive, psychological and social functioning and a lower quality of life score. In the prospective analysis, overall the physical, cognitive and psychological functioning and the quality of life decreased over time. Visual impairment was related to an accelerated deterioration in BADL functioning over time. However, no correlation was found with IADL, cognitive, psychological or social functioning and quality of life. Moreover, the severe visual impaired group had an accelerated decline in BADL and a less decrease in quality of life over time. In addition, mortality risks were the highest in the group with severe visual impairment.

In accordance with previous research [24–26] an association was found between visual impairment and physical functioning in older people. Moreover, the effect of visual impairment on physical functioning was found to be independent of comorbidity and cognitive status [25]. In contrast to our research, studies reported that participants with visual impairment experienced more impairments with IADL activities compared to BADL activities, explained by the requirement to need better visual abilities for instrumental activities [24, 26]. In the present study, the accelerated deterioration solely found in BADL functioning, could be explained by the fact that the participants had already worse IADL functioning and could therefore not clearly change. In addition, another study [13] hypothesized the possible protective role of physical activity against visual impairment and they suggest that the level of physical functioning may be affected by visual impairment. This might explain the results from our in-depth analysis where we found an accelerated deterioration in BADL for the physically impaired group with visual impairment. Thus, visual impairment in combination with a physical impairment at baseline could be a possible predictor for accelerated deterioration in basic activities in daily living.

Moreover, in line with other research, visual impairment was associated with lower cognitive functioning and significantly higher levels of depression [27, 28]. An earlier study by *Gussekloo *et al*.* [16], with these data from the Leiden 85-plus Study, established that older people with visual impairment scored lower on visual cognitive tests, as a consequence of test problems due to their visual impairment. Additionally, in older people with lower cognitive function, the visual impairment could be overestimated because of less understanding of the instruction for the visual test. [16, 29].

Our results presented less decrease in quality of life in the severely visual impaired group. This result was also observed in our in-depth analysis for the people without physical impairment at baseline. Several studies showed that poor physical health, consequently visual impairment, among older people was hardly related to lower life satisfaction or social participation [5, 30, 31]. Visual impairment often results in anxiety and denial, which over the years decreases due to concepts as acceptance and adaption, influencing life satisfaction and thus quality of life [32]. It highlights the importance of the subjectively experienced well-being amongst older people. Increasing quality of life, obtained through adaptation, coping and acceptation, may be crucial to aging successfully [33]. A qualitative study by *Haanes *et al*.* [34] highlights the personal consequences caused by visual impairments in very old adults. Monitoring these people with visual impairment could be crucial to retain their independence and limit social isolation.

Consistent with other research [35], we have established a correlation between severe visual impairment and mortality. Visual impairment often goes hand in hand with more chronic diseases, a lower socio-economic status and an unhealthier lifestyle, all relating to a higher mortality [35].

The Cochrane review by *Clarke *et al*.* [3] indicated that visual screening did not improve vision in people aged 65 years or older, by referring them to suitable healthcare services. However, a systemic review by *Nastasi* [36] highlights the importance of future research to investigate the effects of interventions on visual status. Furthermore, research by van *Nispen *et al*.* [37] showed that basic ophthalmologic screening could help reduce the burden of visual impairment. *Tan CS *et al*.* [38] found that incentive-based intervention schemes increased compliance of attendance after community eye-screening. Thus, establishing the correlation between vision, functioning and mortality could be beneficial to increase the effectiveness of visual screening, by creating more awareness amongst the older people who are visually impaired [5].

The present study has several strengths. Primary, the population-based cohort had no exclusion criteria, an almost complete follow-up and a high response rate, enabling the generalisation of the conclusions to the overall elderly population. Secondly, multiple relevant endpoints, with repeated longitudinal measurements, were used for analysis, including functional status, quality of life and mortality. In addition, the growing aging population highlights the importance of clinical studies, specifically targeting the older population, to increase scientific evidence [36]. Moreover, a review study by *Clarke *et al*.* established that there is no difference between self-reported or identified vision problems by a vision test. Therefore, the standardised measurement of visual acuity was acknowledged as suitable screening tool [3]. A possible limitation of the present research is that only the results of the visual acuity were used for analysis, often resulting in an underestimation of visual impairment [36].

In conclusion, in very old adults, people with visual impairment are at risk for a lower level in physical, cognitive, psychological and social level of functioning. Moreover, visual impairment is associated with a reduced quality of life at baseline and the mortality risks are higher when severely visually impaired. In addition, visual impairment is a predictive factor for accelerated deterioration in physical functioning, mainly for activities in daily living. Highlighting this negative association could induce a pro-active attitude, focusing on preventing and treating visual impairment, possibly helping to improve physical independence, wellbeing and successful aging in very old age. However, more research is needed to establish this contribution.

## Supplementary Information

**Additional file 1.**

## Data Availability

All authors had full access to the study data (including statistical reports and tables) and can take responsibility for the data integrity and the accuracy of the analysis. The datasets analysed during the current study are available from the corresponding author on reasonable request.

## Abbreviations

VA: Visual acuity
ETDRS: Early Treatment Diabetic Retinopathy Study charts
GARS: Groningen Activity Restriction Scale
BADL: Basic activities of daily living
IADL: Instrumental activities of daily living
MMSE: Mini-Mental State Examination
DJG: De Jong Gierveld Loneliness Scale
QoL: Quality of life, HR: hazard ratios
CI: Confidence intervals
ICD-10: International Classification of Diseases and Related Health Problems
