# Supplementary material for: Visual impairment as a predictor for deterioration in functioning: the Leiden 85-plus Study
Source: BMC Geriatr. 2022 May 6;22:397. doi: 10.1186/s12877-022-03071-x (PMC9074345; doi:10.1186/s12877-022-03071-x)
Supplement: Supplementary file 1 — Additional file 1. [file 12877_2022_3071_MOESM1_ESM.pdf]

Additional file 1. Sensitivity analysis according to cut-off values

Description of data: the overall analysis was repeated with different cut-off values for visual impairment; (1) three equal groups according to 33% tertiles (high, medium and low vision), (2) three groups following the International Classification of Diseases and Related Health Problems (ICD-10) guidelines, with a visual acuity below 0.33 defined as low vision and 0.5 or higher as normal vision, (3) decrease per 0.1 point in vision.

Stratified according to tertiles

A sensitivity analysis was done with different cut-off values. When dividing the participants according to tertiles, the same group distribution was formed with identical results as the original analysis.

Stratified according to the ICD-10 guidelines

An overview of the baseline characteristics according to cut-off values, following the ICD-10 guidelines is given in Supplementary table S1. *P* for trend was significant for nearly the same variables as the original analysis, with the exception of male gender (*p* = 0.226) and arthritis (*p* = 0.068). Moreover, Parkinson's disease became significant with a *p* value of 0.045, occurring more often in the group with low vision.

Supplementary table S1. Baseline characteristics, according to levels of vision in ICD-10.

	**All**<br>**N = 548**	**Normal vision**<br>**N = 430**	**Mild vision**<br>**N = 48**	**Low vision**<br>**N = 70**	***P Value****
Demographics and health (No., %)					
Male	184 (33.6)	152 (35.3)	12 (25)	20 (28.6)	0.226
Living arrangements: Independently	462 (84.3)	378 (87.9)	35 (72.9)	49 (70.0)	**< 0.001**
Education > elementary school °	193 (35.2)	154 (35.8)	16 (33.3)	23 (32.9)	0.883
High income °	271 (49.5)	225 (52.3)	18 (37.5)	28 (40.0)	**0.039**
Chronic diseases					
Arthritis/osteoarthritis °	179 (32.7)	150 (34.9)	14 (29.2)	15 (21.4)	0.068
Obstructive pulmonary disease	63 (11.5)	49 (11.4)	5 (10.4)	9 (12.9)	0.911
Cerebrovascular accident °	44 (8.0)	33 (7.7)	6 (12.5)	5 (7.1)	0.458
Myocardial infarction °	56 (10.2)	43 (10.0)	4 (8.3)	9 (12.9)	0.695
Parkinson's disease	11 (2.0)	7 (1.6)	0 (0.0)	4 (5.7)	**0.045**
Malignancy °	100 (18.2)	77 (17.9)	10 (20.8)	13 (18.6)	0.869
Diabetes mellitus °	79 (14.4)	50 (11.6)	13 (27.1)	16 (22.9)	**0.001**
Severe cognitive impairment (MMSE < 19) °	89 (16.2)	56 (13.0)	12 (25.0)	21 (30.0)	**< 0.001**
History fall °	93 (17.2)	72 (16.9)	11 (22.9)	10 (14.8)	0.659
Hip fracture °	33 (6.1)	21 (4.9)	3 (6.4)	9 (12.9)	**0.012**
Functioning and quality of life (median, IQR)					
Physical functioning:					
BADL (n=547)	10.0 (9.0-14.0)	9.0 (9.0-12.0)	11.0 (9.0-16.8)	13.0 (9.0-13.0)	**< 0.001**
IADL (n=547)	18.0 (12.0-25.0)	16.0 (12.0-23.0)	22.0 (12.3-31.8)	24.0 (16.0-34.0)	**< 0.001**
Cognitive functioning: MMSE	26.0 (23.0-28.0)	27.0 (24.0-28.0)	25.5 (20.3-28.0)	24.0 (18.0-28.0)	**< 0.001**
Psychological functioning: GDS (n=475) #	2.0 (1.0-3.0)	2.0 (1.0-3.0)	2.0 (1.0-4.0)	3.0 (1.0-4.8)	**0.001**
Social functioning: DJG (n=476) #	1.0 (0.0-3.0)	1.0 (0.0-2.0)	2 (0.0-4.3)	2.0 (1.0-3.0)	**< 0.001**
Quality of Life: Cantril (n=521)	8.0 (7.0-9.0)	8.0 (7.0-9.0)	7.0 (6.0-8.0)	7.0 (6.0-8.5)	**0.016**

BADL, Basic Activities Daily Living (range 9-36); IADL, Instrumental Activities Daily Living (range 9-63); MMSE, Mini Mental State Examination (range 0-30); GDS, Geriatric Depression Scale (range 0-15); DJG, De Jong Gierveld Loneliness Scale (range 0-11); Cantril, Cantril's ladder of life (range 0-10); IQR, Interquartile Range. The median and interquartile ranges are provided when continuous variables have an asymmetric distribution. For categorical variables percentages are presented. **P Value* for between group comparison with regard to vision, measured with *P for trend*: for categorical data with Linear-by-Linear Association, for continuous data with Jonckheere-Terpstra Test. ° Missing data for specific variables, according to three groups (severe – moderate – no impairment): 0-7 missing. #Assessed only in participants with MMSE > 18.

A linear association was found for the level of functioning and time ($p < 0.001$), however, for the Loneliness scale the basic annual change correlation was lacking ($p = 0.873$). The additional annual change for BADL in the group with low vision was leaning towards a trend with an increase of 0.33 points, however, not significant ($p = 0.058$, SE 0.174). Moreover, low vision was associated with a significant additional annual increase of 0.13 points for the Cantril's Ladder ($p = 0.028$, SE 0.059). The effect of visual impairment on the level of functioning and quality of life is demonstrated in Supplementary table S2.

Supplementary table S2. Effect of visual impairment on functioning and quality of life, ICD-10.

	Basic annual change			Additional annual change					
	Normal vision			*Mild vision*			*Low vision*		
	β_1	SE	*P Value*	β_2	SE	*P Value*	β_2	SE	*P Value*
BADL	1.22	0.056	**< 0.001**	0.09	0.199	0.660	0.33	0.174	0.058
IADL	2.26	0.058	**< 0.001**	0.11	0.207	0.579	-0.32	0.181	0.080
MMSE	-0.75	0.038	**< 0.001**	0.09	0.135	0.492	-0.10	0.117	0.397
GDS	0.30	0.029	**< 0.001**	-0.13	0.104	0.219	0.03	0.099	0.748
DJG	0.03	0.023	0.126	-0.15	0.078	0.063	-0.06	0.074	0.421
Cantril	-0.21	0.017	**< 0.001**	0.10	0.063	0.123	0.13	0.059	**0.028**

SE, standard error. *P Values* were estimated by analysis of linear mixed models, significant when *P Value* < 0.05; function of the linear mixed model according to: $y = \alpha + \beta x$. β firstly represents the basic annual change over time without impairment; and secondly the additional annual change for people with visual impairment. β is given with corresponding SE.

Cross-sectional correlation

Analysis of the cross-sectional effect showed that BADL decreased with 5.83 points when vision increased with one unit. This effect was statistically significant with a p value < 0.001. However, solely 7.7% of the variance in vision can be explained by functional status. Significant patterns were found for IADL, MMSE, GDS and DJG ($p < 0.001$). Furthermore, quality of life increased with 1.03 points when vision rose with one unit ($p = 0.001$). Results are depicted in Supplementary table S3.

Supplementary table S3. Cross-sectional effect of decline in vision per point on level of functioning.

	**β**	SE	R^2	*P Value*
Basic Activities Daily Living	-5.83	0.867	0.077	**< 0.001**
Instrumental Activities Daily Living	-11.62	1.400	0.112	**< 0.001**
Mini Mental State Examination	6.30	0.875	0.087	**< 0.001**
Geriatric Depression Scale	-2.12	0.464	0.042	**< 0.001**
De Jong Gierveld Loneliness Scale	-1.51	0.421	0.026	**< 0.001**
Cantril's Ladder	1.03	0.317	0.020	**0.001**

SE, standard error. *P Values* were estimated using linear regression analysis, significant when *P Value* < 0.05; function of the linear mixed model according to: $y = \alpha + \beta x$. β is the unstandardized β and it refers to the number of units the functional assessment tools increase for a single unit increase in vision. β is given with corresponding SE. R^2 indicates the proportion of variance in vision that can be explained by the functional levels.
